# Using bioelectrohydrogenesis left-over residues as a future potential fertilizer for soil amendment

**DOI:** 10.1038/s41598-022-22715-x

**Published:** 2022-10-22

**Authors:** Fabrice Ndayisenga, Zhisheng Yu, Bobo Wang, Jie Yang, Gang Wu, Hongxun Zhang

**Affiliations:** 1 College of Resources and Environment, University of Chinese Academy of Sciences, 19 A Yuquan Road, Beijing, 100049 People’s Republic of China; 2 Binzhou Institute of Technology, Weiqiao-UCAS Science and Technology Park, Binzhou, 256606 Shandong People’s Republic of China; 3 RCEES-IMCAS-UCAS Joint-Lab of Microbial Technology for Environmental Science, Beijing, 100085 People’s Republic of China; 4 State Key Laboratory of Urban and Regional Ecology, Research Center for Eco-Environmental Sciences, Chinese Academy of Sciences, Beijing, 100085 People’s Republic of China

**Keywords:** Biogeochemistry, Ecology, Environmental sciences

## Abstract

In this current research, the left-over residues collected from the dark fermentation-microbial electrolysis cells (DF-MEC) integrated system solely biocatalyzed by activated sludge during the bioconversion of the agricultural straw wastes into hydrogen energy, was investigated for its feasibility to be used as a potential alternative biofertilizer to the commonly costly inorganic ones. The results revealed that the electrohydrogenesis left-over residues enriched various plant growth-promoting microbial communities including *Enterobacter* (8.57%), *Paenibacillus* (1.18%), *Mycobacterium* (0.77%), *Pseudomonas* (0.65%), *Bradyrhizobium* (0.12%), *Azospirillum* (0.11%), and *Mesorhizobium* (0.1%) that are generally known for their ability to produce different essential phytohormones such as indole-3-acetic acid/indole acetic acid (IAA) and Gibberellins for plant growth. Moreover, they also contain both phosphate-solubilizing and nitrogen-fixing microbial communities that remarkably provide an adequate amount of assimilable phosphorus and nitrogen required for enhanced plants or crop growth. Furthermore, macro-, and micronutrients (including N, P, K, etc.) were all analyzed from the residues and detected adequate appreciate concentrations required for plant growth promotions. The direct application of MEC-effluent as fertilizer in this current study conspicuously promoted plant growth (*Solanum lycopersicum *L. (tomato), *Capsicum annuum* L. (chilli), and *Solanum melongena* L. (brinjal)) and speeded up flowering and fruit-generating processes. Based on these findings, electrohydrogenesis residues could undoubtedly be considered as a potential biofertilizer. Thus, this technology provides a new approach to agricultural residue control and concomitantly provides a sustainable, cheap, and eco-friendly biofertilizer that could replace the chemical costly fertilizers.

## Introduction

The excessive production of biodegradable wastes from the agricultural sectors can inevitably harm our living environment if they are not adequately controlled. The anaerobic fermentation of wastes for biogas and/or other bioactive molecule production is of great interest for both agricultural waste management and energy recovery^1^. Anaerobic fermentation counts a number of beneficial features including the production of renewable energy, reduction of greenhouse gas emissions, and alleviation of the gravity of the agricultural wastes-driving detrimental issues^2,3^. Though the fermentation process is considered as a promising strategy to control agricultural wastes, it generates along with biogas, the fermentation residues (commonly known as digestate) that could intensify the problem of environmental pollution if it is not well addressed. Therefore, its adequate management should be considered to ensure the implementation of anaerobic fermentation technology on a large -scale.

Prior to waste management, Directive et al. proved that fermentation effluent can be used as a soil quality booster, which could lead to agricultural or ecological improvements, and it has been adopted as an appropriate approach. However, to ensure the sustainable recycling of the fermentation residues through agriculture, their composition features, stability, and hygiene should be characterized before use^4^. Generally, digestate enriches various nutrients and can be favorably selected over commercial inorganic fertilizers for promoting crop yield, productivity, and soil quality^5,6^. Moreover, digestate was reported to contain higher nutrient content than its producing substrate. Even though a huge amount of nitrogen is emitted as ammonium (NH_4_) during the fermentation process and carbon is removed in the form of both methane and CO_2_, it still remains a reasonable amount of N, phosphorous (P), and potassium (K) in the fermentation residues^7^. Therefore, fermentation effluent could play beneficial effects on soil quality and/or plant health.

It holds fertilizing traits that remarkably promote plant productivity owing to the availability of essential nutrients for plant growth. Digestive residues can also play a major role in promoting soil efficiency via carbon transformation, soil nutrient cycling, and the maintenance of the soil structure^8^. They noticeably contribute to the availability of soil N, which is considered as a key essential element promoting plant growth and metabolic activities of the soil microbial communities. Nitrogen is largely uptaken by plants/crops and therefore taken as the major limiting factor for plant development^9,10^. Moreover, fermentation effluent was also reported to be a potential source of ammonium nitrogen readily uptaken by plants as well^11^.

On the other hand, the fermentation residues enriched various microbial communities, and once applied to seeds or plants colonize the rhizosphere which thus results in promoting plant growth by augmenting nutrient supply to the hostplant^12,13^. These additional fertilizing attributes of the digestive residues imply their great possibility to be employed as a potential biofertilizer. Biofertilizers are largely used to speed up microbial metabolic activities leading to the increment of the easily-assimilable nutrient availability for the plant and/or crop. Moreover, they generate plant growth-promoting materials, solubilize insoluble phosphates in the soil, and ameliorate soil fertility via atmospheric nitrogen fixation^14^. Therefore, based on those aforementioned fertilizing features of the fermentation residues, it could undoubtedly improve the soil quality once applied as a biofertilizer. However, the occurrence of nitrogen-fixing bacteria, mineral contents, and characteristics of digestate depend on the nature of the substrate and the modality of digestion^15^.

**Figure 1:**
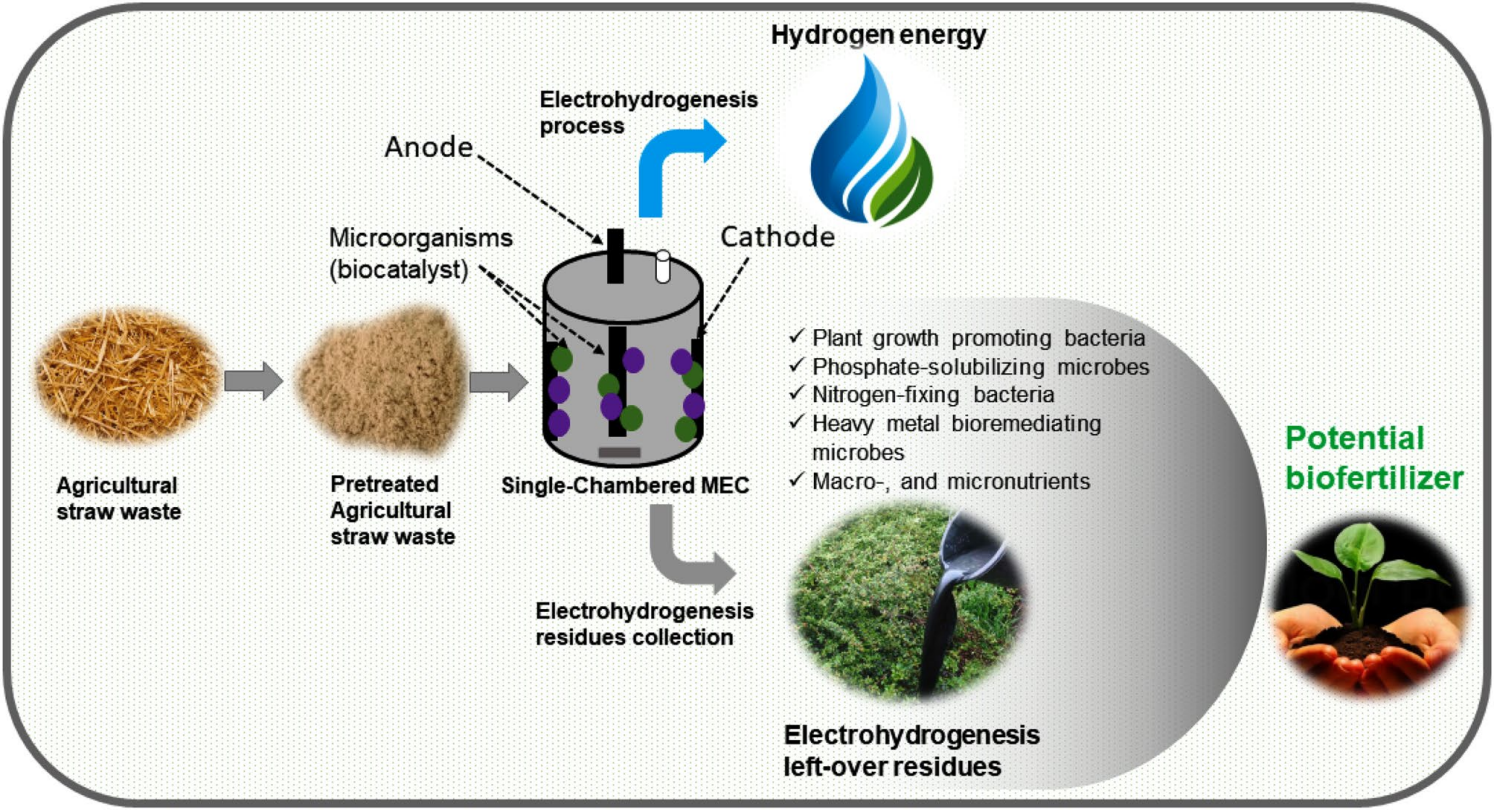
Schematic diagram illustrating the general concept of using the straw waste-fed MEC effluent as a potential biofertilizer.

Though, it seems massive research works exploring the feasibility of using digestate from the normal livestock anaerobic digestion process as fertilizers have been done, but up-to-now there is no study done investigating the possibility of using digestate resulting from the bio-electrohydrogenesis process as fertilizer. This novel work aims to characterize and investigate the possibility of using the effluent residues from the microbial electrolysis cells combined with the dark fermentative process during the conversion of the lignocellulosic agricultural wastes into biohydrogen energy as a novel potential fertilizer (see Fig. 1). Both electrohydrogenesis residue's nutritional composition and associated plant growth-promoting bacterial communities were all analyzed and reported, to ensure its high fertilizing-capacity attributes. Moreover, the collected MEC-effluent was directly used as fertilizer to grow three selected crops namely *Solanum lycopersicum L* (tomato), *Capsicum annuum L.* (chilli), and *Solanum melongena L.* (brinjal).

## Materials and methods

### Electrohydrogenesis effluent-producing process and collection

Herein, the employed residues resulted from the dark fermentation and microbial electrolysis cell integrated system operated for the bioconversion of agricultural wastes into biohydrogen energy in the process bio-catalyzed by microorgafirnisms (in the form of activated sludge) obtained from the UCAS Yanqi campus sewage treatment plant, Beijing, China. The used agricultural wastes served as carbon sources, was the air-dried wheat straw waste obtained from Wei County, Xingtai City, Hebei Province, China. Prior to use, the washed biomass was dried by the oven (101-3AB, Tianjin Taintai Yiqi Youxian Co., Ltd., China) at 105 °C for 1 h; and then chopped and pulverized by a crushing instrument (FE130, Staida Co., Tianjin, China). The resulting straw powder was then mixed with dilute H_2_SO_4_ (0.5%) at a ratio of 1:10 (solid to liquid) and then hydrolyzed at 121 °C for 60 min. The hydrolyzed mixture was then neutralized to pH 7.0 ± 2 with dilute Ca(OH)_2_ solution and then served as an electron donor during the electrohydrogenesis process^16^.

The electrohydrogenesis process was performed in a 3 L single-chambered MEC reactor, using a carbon brush (porosity was 95%) and carbon cloth covered by stainless steel mesh (30 mesh) as anode and cathode electrodes respectively. The electrodes were arranged within reactors as previously reported by Yan et al.^17^. The reactor operating temperature was set at 55 °C and performed for 24 days. At the end of the MEC performance, the effluent residues were then collected and investigated in this current report for their possibility to be considered as a potential alternative biofertilizer to the costly inorganic fertilizer, instead of leaving it in the open environment where they can cause other detrimental problems (see Fig. 1).

### Determination of MEC-effluent composition

To ensure the fertilizing attributes of the DF-MEC integrated residues, the nutritional composition characterized by total organic carbon (TOC), and total nitrogen (TN) were analyzed based on the standard methods^18^. Prior to further nutrients analysis, the collected sample was hydrolyzed in nitric acid perchloric acid (HNO_3_ 3%) and the macro-and micronutrients (P, K, Ca, Na, Mg, and S), as well as heavy metals (Mn, Ni, Cu, Zn, and Pb), were then detected by Inductively coupled plasma-mass spectrometry (XSERIES 2 ICP-MS; Thermo Scientific, MA, USA) as previously reported^19^.

### Microbial community analysis

The samples were collected from DF-MEC integrated reactors’ effluent and the microbial community analysis was performed by Major Bio. Co., Ltd. (Shanghai, China) by employing polymerase chain reaction amplification and high-throughput sequencing technology which were performed by Major Bio. Co., Ltd. A rapid Power Soil DNA Isolation Kit (Mo Bio Laboratories, Inc, USA), was used to extract biofilm genomic deoxyribonucleic acid (DNA), according to the manufacturer’s instructions. Miseq sequencing was constructed for Illumina, using bacterial primers 338F (5′-ACTCCTACGGGAGGCAGCAG-3′) and 806R (5′-GGACTACHVGGGTWTCTAAT-3′) for the V3–V4 region of the 16S rRNA gene^20^. To minimize the effects of random sequencing errors, low-quality sequences were removed, by eliminating those without an exact match with the forward primer, those without a recognizable reverse primer, length shorter than 200 nucleotides, or longer than 460 bp, and containing any ambiguous base calls (Ns). The remaining sequences were performed as previously reported by Yang et al. and Caporaso et al.^21,22^, and the similar sequences were clustered into operational taxonomic units with a 97% similarity employing USEARCH^23^, and the related species diversity and abundance were analyzed to find the dominant species in the investigated protocols.

### Plant growth experiments

Eighteen plastic trays (16.5 cm (diameter) × 15 cm (height)) were bought and used in this plant growth experiment as soil containers. The trays were filled with the same amount of soil (~ 4 kg for each), and 9 of them were supplied with the electrohydrogenesis effluent whereas the remaining 9 were supplied only with water (without MEC effluent) and marked as controls. The cultivated crops were tomato (*Solanum lycopersicum* L.), chilli (*Capsicum annuum* L.), and brinjal (*Solanum melongena* L.), and their plantlets were purchased from Beijing Shengke Oriental Technology Co., LTD, Beijing, China. None of the selected crop species were on the IUCN (International Union for Conservation of Nature) Red List of threatened species and all conducted procedures in this current study never violated international guidelines and regulations for protecting plant species at high risk of extinction as published by both IUCN Policy Statement on Research Involving Species at Risk of Extinction and Convention on International Trade in Endangered Species of Wild Fauna and Flora.

Before planting them onto their respective trays, they first got washed off the primitive soil to free the roots for increasing the contact surface area for our prepared soil. Each single species was planted in triplets in soil with MEC-effluent (Soil + Effluent) to avoid growth data errors and directly compared with its corresponding plant grown in soil with water (Soil + Water). To avoid soil dryness during cultivation, the electrohydrogenesis effluent liquid and water (~ 0.5 L) were regularly added every 5 days to their respective protocols, and the plant height and number of leaves were monitored over one month at an interval of 5 days as well. At the end of the 3rd month of the cultivation, the crop growth was evaluated in times of the fruit yield, by accounting for fruit number and size per crop (plant), and got directly compared to their corresponding controls. The experimental setup is summarized in Table 1.

**Table 1:** Experimental setup for plant cultivation.

Protocols	Plantlets	Cultivation period (day (D))																		
		Watering volume (electrohydrogenesis effluent or water) (L)																		
		1D	5D	10D	15D	20D	25D	30D	35D	40D	45D	50D	55D	60D	65D	70D	75D	80D	85D	90D
Soil + effluent	Tomato	0.5	0.5	0.5	0.5	0.5	0.5	0.5	0.5	0.5	0.5	0.5	0.5	0.5	0.5	0.5	0.5	0.5	0.5	0.5
	Chilli	0.5	0.5	0.5	0.5	0.5	0.5	0.5	0.5	0.5	0.5	0.5	0.5	0.5	0.5	0.5	0.5	0.5	0.5	0.5
	Brinjal	0.5	0.5	0.5	0.5	0.5	0.5	0.5	0.5	0.5	0.5	0.5	0.5	0.5	0.5	0.5	0.5	0.5	0.5	0.5
Soil + water	Tomato	0.5	0.5	0.5	0.5	0.5	0.5	0.5	0.5	0.5	0.5	0.5	0.5	0.5	0.5	0.5	0.5	0.5	0.5	0.5
	Chilli	0.5	0.5	0.5	0.5	0.5	0.5	0.5	0.5	0.5	0.5	0.5	0.5	0.5	0.5	0.5	0.5	0.5	0.5	0.5
	Brinjal	0.5	0.5	0.5	0.5	0.5	0.5	0.5	0.5	0.5	0.5	0.5	0.5	0.5	0.5	0.5	0.5	0.5	0.5	0.5

### Statistical analysis methods

This investigation employed ORIGIN software _v9.sr2 and Microsoft Excel for basic descriptive statistics, data treatment, and figure drawings. Data were collected in triplets to minimize statistical errors during data analysis and interpretation.

## Results

### Electrohydrogenesis effluent as a potential biofertilizer

**Figure 2:**
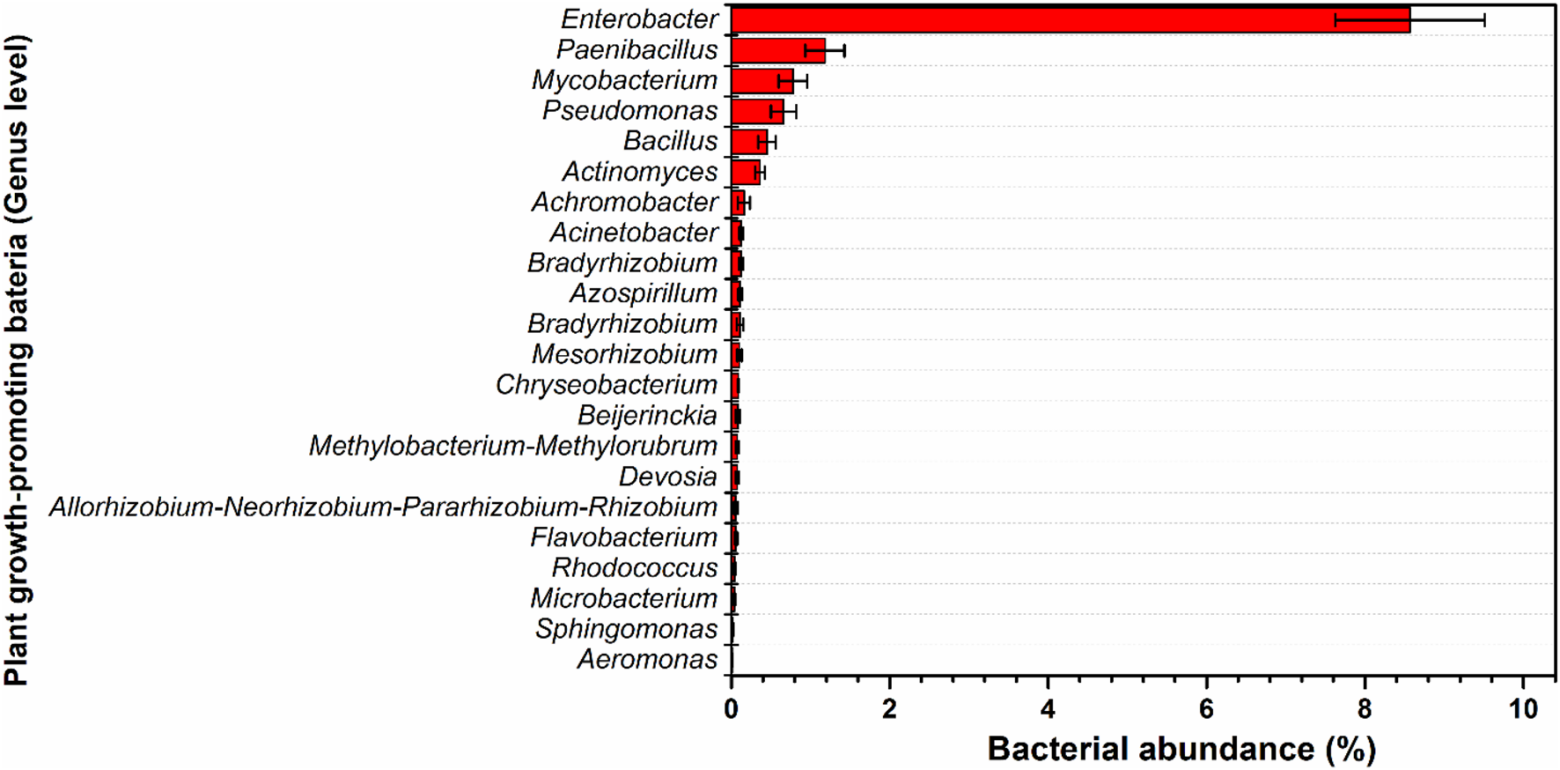
The abundance of the Plant growth-promoting bacteria (genus level) detected from the DF-MEC digestate (%).

To characterize the electrohydrogenesis left-over residues as potential biofertilizers, the sample from the operating reactors was performed a 16S rRNA sequencing test, and interestingly, the results revealed that the bio-electrohydrogenesis effluent was enriched with various microorganisms including plant growth-promoting microbes that display biofertilizer-like features. Among the well-known plant-promoting bacterial genera observed in DF-MEC residues included *Azospirillum*, *Mycobacterium*, *Chryseobacterium*, *Paenibacillus*, *Rhizobacter*, *Pseudomonas, Achromobacter*, *Bradyrhizobium*, *Actinomyces*, *Sphingomonas*, *Allorhizobium-Neorhizobium-Pararhizobium-Rhizobium*, *Gordonia, Rhodococcus*, *Bacillus*, *Methylobacterium-Methylorubrum*, *Microbacterium*, *Flavobacterium*, *Devosia*, *Acinetobacter*, *Mesorhizobium*, *Enterobacter*, *Aeromonas*, *Beijerinckia,* etc.^24–26^ (Fig. 2). Lots of investigations working on the feasibility of using biofertilizers other than chemical fertilizers have revealed that those aforementioned microbes play a major role in providing the required nutrients for enhanced crop yield.

### Nitrogen-fixing microorganisms

**Figure 3:**
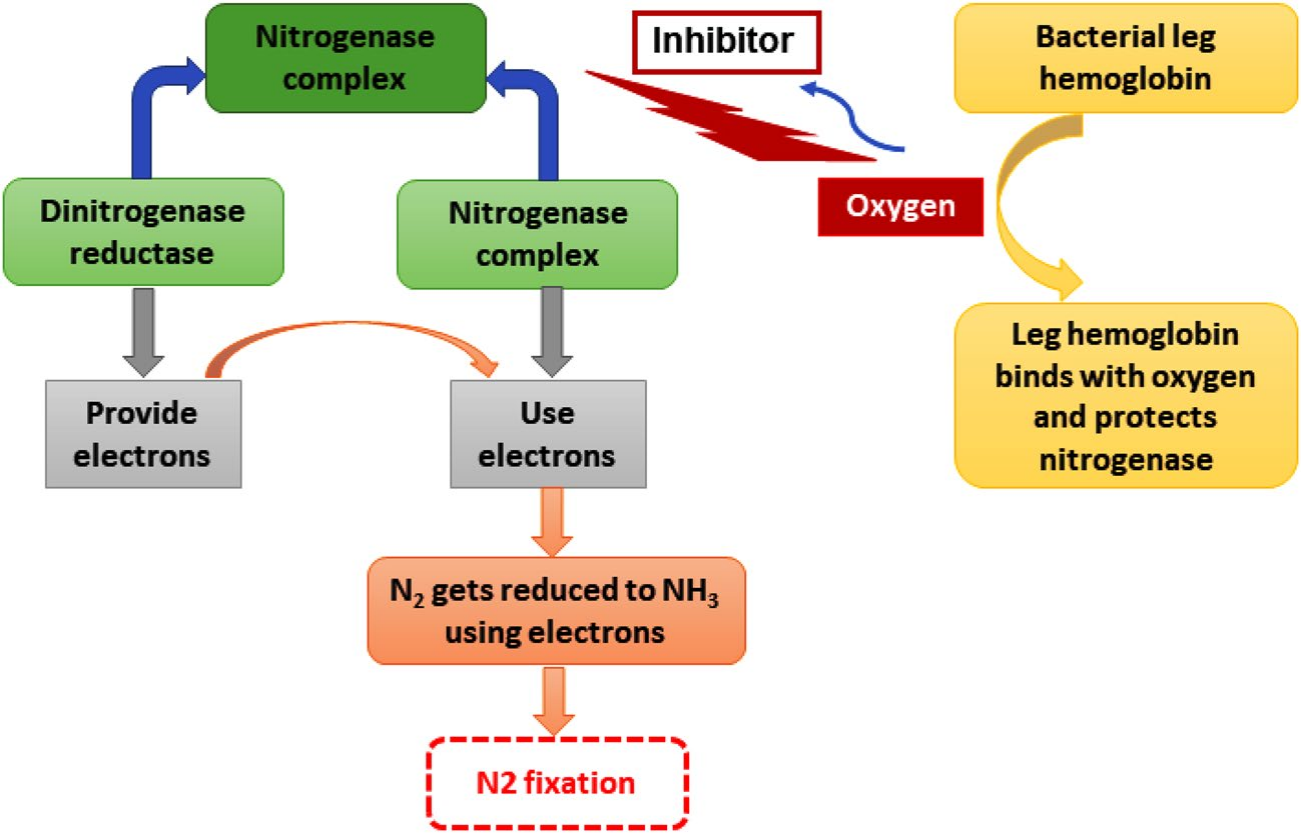
Mechanism of nitrogen fixation bio-catalyzed by nitrogenase enzyme. The plant growth-promoting bacteria produce nitrogenase which is a complex enzyme consisting of dinitrogenase reductase and dinitrogenase. This complex enzyme plays a major role in molecular N_2_ fixation. Dinitrogenase reductase provides electrons and dinitrogenase uses those electrons to reduce N_2_ to NH_3_. However, oxygen is a potential threat to this process since it has the ability to get bound to the enzyme complex and make it inactive and consequently inhibit the process. Interestingly, bacterial leghemoglobin has a strong affinity for O_2_ and thus gets bound to free oxygen more strongly and effectively to suppress the available oxygen effects on the whole process of nitrogen fixation.

The detected nitrogen-fixing microorganisms from the electrohydrogenesis effluent include *Azospirillum* sp. (0.11 ± 0.02%)*,* rhizobia (*Rhizobium (0.058* ± 0.02*%), Bradyrhizobium* (0.11 ± 0.04%), and *Mesorhizobium* (0.1 ± 0.03%)), and *Beijerinckia* (0.08 ± 0.03%) (Fig. 2) and were repeatedly reported for their superior contribution to the plants’ nitrogen requirements through biological nitrogen fixation, which is an important component of sustainable agriculture^25^. Although the atmosphere counts about 78% N_2_, it couldn’t be used by plants in its natural state. Prior to getting used by plants, it needs to be converted to ammonia, which is the readily assimilable form of nitrogen by plants/or crops via a biological nitrogen fixation mechanism^25^. The biological Nitrogen fixation mechanism is summarized in Fig. 3.

### Phosphate-solubilizing microorganisms

**Figure 4:**
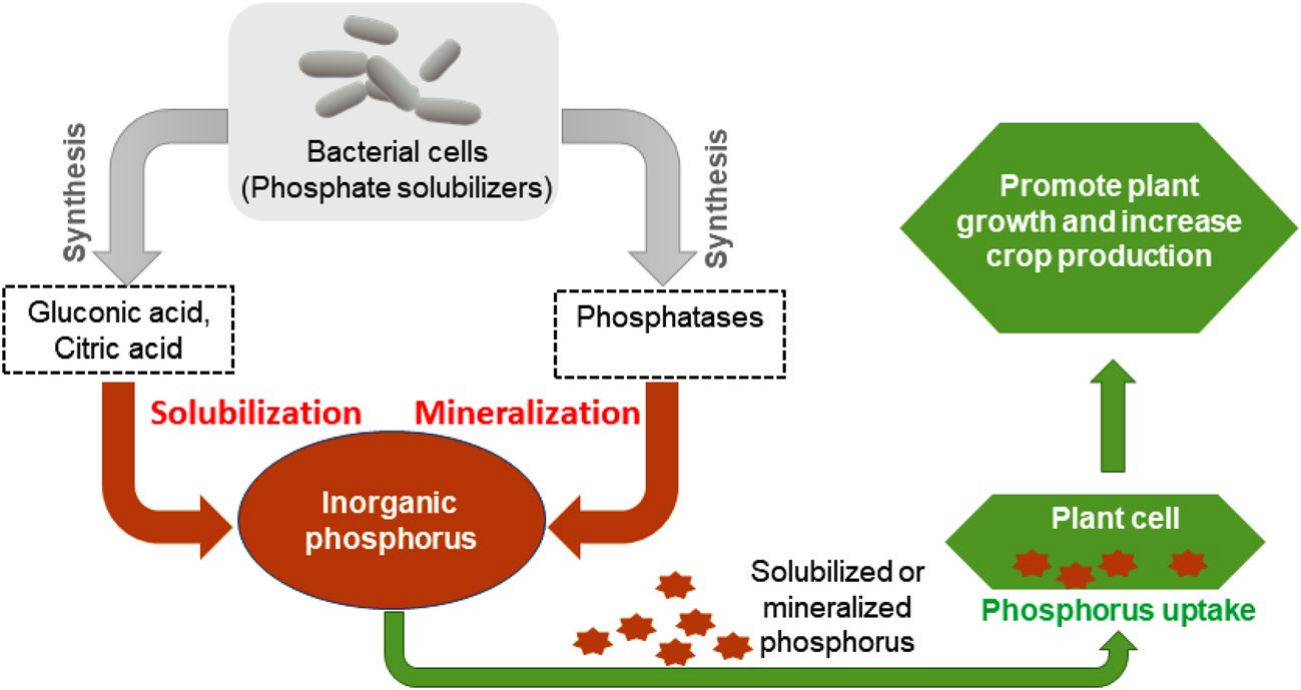
Inorganic phosphorus solubilization by phosphate-solubilizing rhizobacteria. A bacterium solubilizes inorganic phosphorus through the action of low molecular weight organic acids such as gluconic and citric acids. The hydroxyl (OH) and carboxyl (COOH) groups of these acids chelate the cations bound to phosphate and thus convert insoluble phosphorus into a soluble organic form. The mineralization of soluble phosphorus occurs by synthesizing different phosphatases which catalyze the hydrolysis process. When plants incorporate these solubilized and mineralized phosphorus molecules, eventually, overall plant growth and crop yield significantly increase.

Furthermore, various phosphate-solubilizing and mineralizing strains were also found in bioelectrohydrogenesis residues collected from our DF-MEC integrated reactors. Among those microorganisms with the ability to solubilize/metabolize the insoluble inorganic phosphorus, the dominant bacterial genera included *Pseudomonas* (0.65 ± 0.15*%)*, *Bacillus* (0.44 ± 0.11%), *Rhodococcus* (0.04 ± 0.009%)*, Rhizobium* (0.05 ± 0.02%), *Microbacterium* sp. (0.04 ± 0.01%)*, Achromobacter* (0.16 ± 0.07%), and *Flavobacterium* (0.058 ± 0.014%) (Fig. 2). Though enormous amounts of phosphorus are available in the soil, its high portion never contributes to plant growth in its primitive state, unless it is bio-transformed into absorbable forms including monobasic and dibasic. Microbial phosphate solubilizing mechanisms are well described in Fig. 4.

### Phytohormone-producing microorganisms

In this current work, the electrohydrogenesis effluent also contained bacterial genera such as *Mycobacterium* (0.77 ± 0.18%), *Allorhizobium* (0.05 ± 0.02%), *Pararhizobium* (0.05 ± 0.02%), *Paenibacillus* (1.18 ± 0.24%), *Bradyrhizobium* (0.11 ± 0.04%), *Rhizobium* (0.05 ± 0.02%), *Acinetobacter* (0.14 ± 0.02%), and *Azospirillum* (0.11 ± 0.025%) (Fig. 2) that have the ability to synthesize indole-3-acetic acid/indole acetic acid (IAA) through indole-3-pyruvic acid and indole-3-acetic aldehyde^25^. IAA is a well-known type of phytohormone that enhances plant/crop growth. Particularly, *Azospirillum* sp., also produce various phytohormones namely cytokinins, gibberellins, ethylene, abscisic acid and salicylic acid, auxins, vitamins such as niacin, pantothenic acid, and thiamine. The conceptional model delineating the positive effects of inoculation with *Azospirillum* sp*.* a phytohormones-producer plant growth-promoting rhizobacteria and its detailed functions on plant growth are summarized and illustrated in Fig. S1. Therefore, the existence of those rhizobacteria in the bioelectrohydrogenesis residues further implies the suitability of considering the DF-MEC left-over residues as potential biofertilizers.

### Heavy metals-bioremediating microorganisms

Some other bacterial genera with the ability to bioremediate the heavy metal toxicity were also found within the bioelectrohydrogenesis left-over residues as well. Among the detected plant growth-promoting bacterial genera; *Rhizobium* (0.058 ± 0.023%), *Mesorhizobium* (0.1 ± 0.026%), *Bradyrhizobium* (0.11 ± 0.04%), *Pseudomonas* (0.65 ± 0.15%), and *Achromobacter* (0.16 ± 0.077%) were reported for their key contribution to alleviate the toxicity of the heavy metals via bioremediation process and improve the soil quality for a relief plant development^26^ (Fig. 2). Other detected heavy metals-bioremediating microorganisms’ species were *Chryseobacterium sp.* (0.08 ± 0.007%), *Azospirillum* (0.11 ± 0.02%), *Bacillus* (0.44 ± 0.11%), *Enterobacter* (8.57 ± 0.9%), *Gordonia* (0.06 ± 0.02%), *Paenibacillus* (1.18 ± 0.24%), *Pseudomonas* (0.65 ± 0.15%), and *Actinomycetes* (0.36 ± 0.05%) that either use microbial siderophores or enzymatic biodegradation process.

### Electrohydrogenesis left-over residues as a potential source of essential elements for plant growth

**Figure 5:**
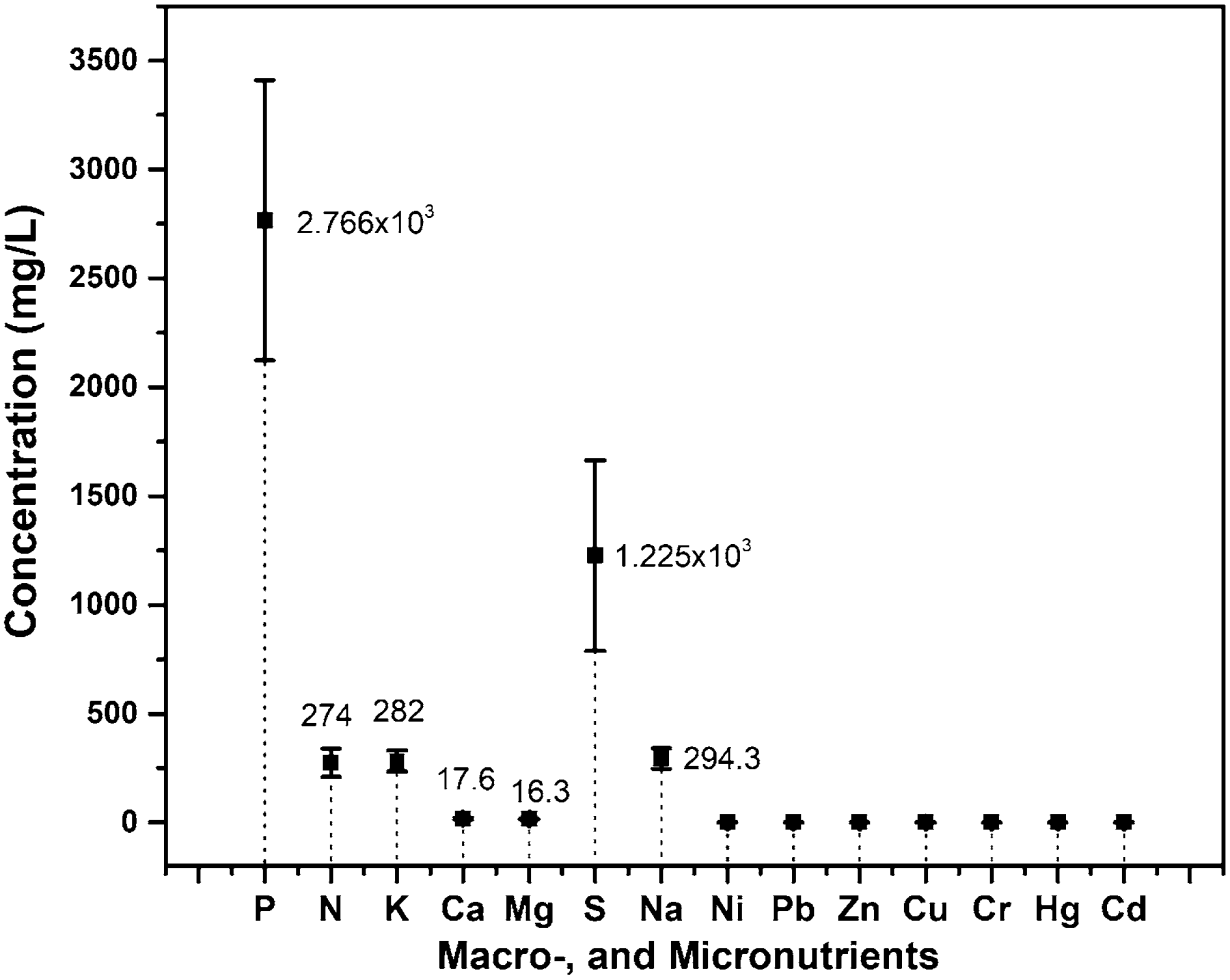
Macro-, and micronutrients detected from the bio-electrohydrogenesis left-over residues (mg/L).

As aforementioned in “Materials and methods” section, the electrohydrogenesis left-over residues contained diverse microbial communities that degraded the MEC substrate and generate biogas and inorganic compounds. Moreover, it has been reported that those inorganic nutrients are generally available in fermentation effluent in readily plant-utilizable formats owing to substrate mineralization^27^. Beside detecting various plant growth-promoting microorganisms in the electrohydrogenesis effluent, a larger number of mineral elements essential for promoted growth and development of crop plants were also investigated and analyzed from the residues. The detected primary and secondary macro-elements’ concentrations in the residues were arranged in decreasing order as follows P > S > Na > K > N > Ca > Mg. Interestingly the findings show that the residues abundantly contained Phosphorus (2.766 × 10^3^ mg/L), Nitrogen (274 mg/L), Potassium (282 mg/L), Calcium (17.66 mg/L), Magnesium (16.3 mg/L), Sulfur (1.225 × 10^3^ mg/L), and Sodium (294.3 mg/L) which are well known as macro-nutrients needed in larger amounts for enhanced plant/ crop growth (Fig. 5).

Moreover, small amounts of the microelements including Ni, Pb, Zn, Cu, Cr, Hg, Cd were also found in the electrohydrogenesis residues, and consistently these elements are generally required in small quantities for the development of plants (Fig. 5), otherwise, their high concentrations are toxic for the plant cells thus suppress or inhibit plant growth. The detected concentrations for the main microelements in this current research ranged only from 0.36 to 9.6 × 10^–5^ mg/L and were all reported to play fundamental roles in plant metabolic reactions.

### Cultivation of the leguminous crops using electrohydrogenesis left-over residues as fertilizer

**Figure 6:**
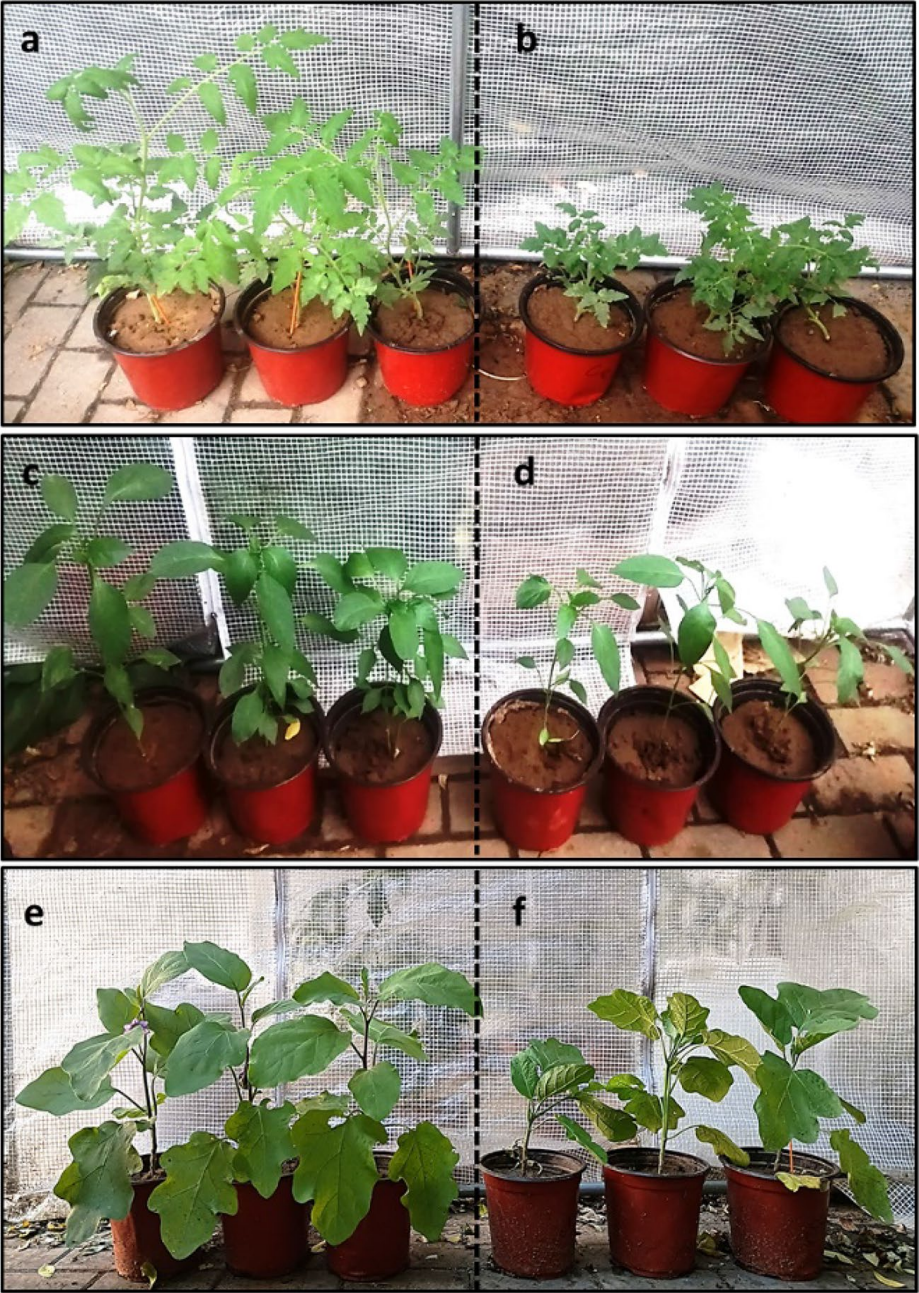
Analysis of the plant growth at the end of the 1st month of cultivation. (**a**) Tomato in soil with effluent, and its control without effluent (**b**); (**c**) Chilli grown in soil with effluent, and its control without effluent (**d**); and (**e**) brinjal grown in soil with effluent, and its corresponding control grown without effluent (**f**) (after 2 months).

After evaluating the plant-growth promoting bacterial communities and the macro- and micronutrients required for plant/crop growth in the electrohydrogenesis left-over residues, the latter was directly used as fertilizer to grow three different plant species including tomato, chili, and brinjal as afore-described in the “Materials and methods” section. To access the potentials of the electrohydrogenesis effluent as fertilizer, the plants grown in the soil amended with the effluent (Soil + Effluent), were directly compared with their corresponding control plants (Soil + water). The results indicated that at the end of 1st month, the plants with effluent grew faster and generated a good amount of branching than the control plants (see Fig. 6), possibly due to the availability of both microbial species with bio-fertilizing aspects and micro-and macronutrients in the effluent.

**Figure 7:**
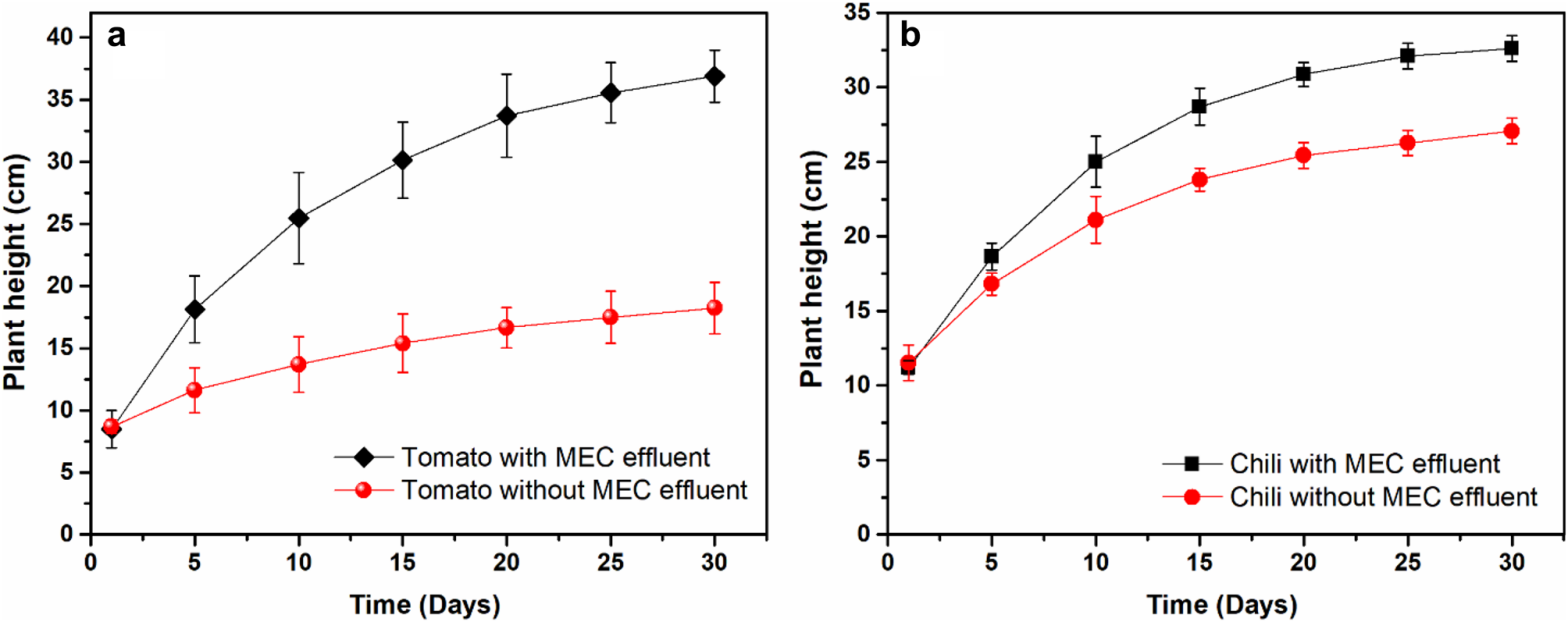
Daily plant growth analysis within one month of cultivation. (**a**) Tomato growth monitoring, (**b**) Chili growth analysis.

For instance, tomato (*Solanum lycopersicum* L.) and chilli (*Capsicum annuum* L.) height in soil + electrohydrogenesis effluent was ~ 36.9 ± 2.1 cm and ~ 32.6 ± 0.8 cm respectively which was ~ 2.03 and ~ 1.2 times the height of their corresponding plant species in the control protocol, respectively (see Fig. 7). However, the brinjal species (*Solanum melongena* L.) didn’t show any remarkable height differences in both protocols after a month of cultivation (data not shown), probably due to their low adaptative characteristics to the new environment. However, after the 2nd month, the brinjal height in soil + effluent became 2.7 times that of the brinjal control cultivated without effluent (see Fig. 6e,f). Moreover, both the number of the plants' leaves and their length in plants cultivated in soil + effluent, were remarkably higher than in plants grown without the supply of the effluent.

**Figure 8:**
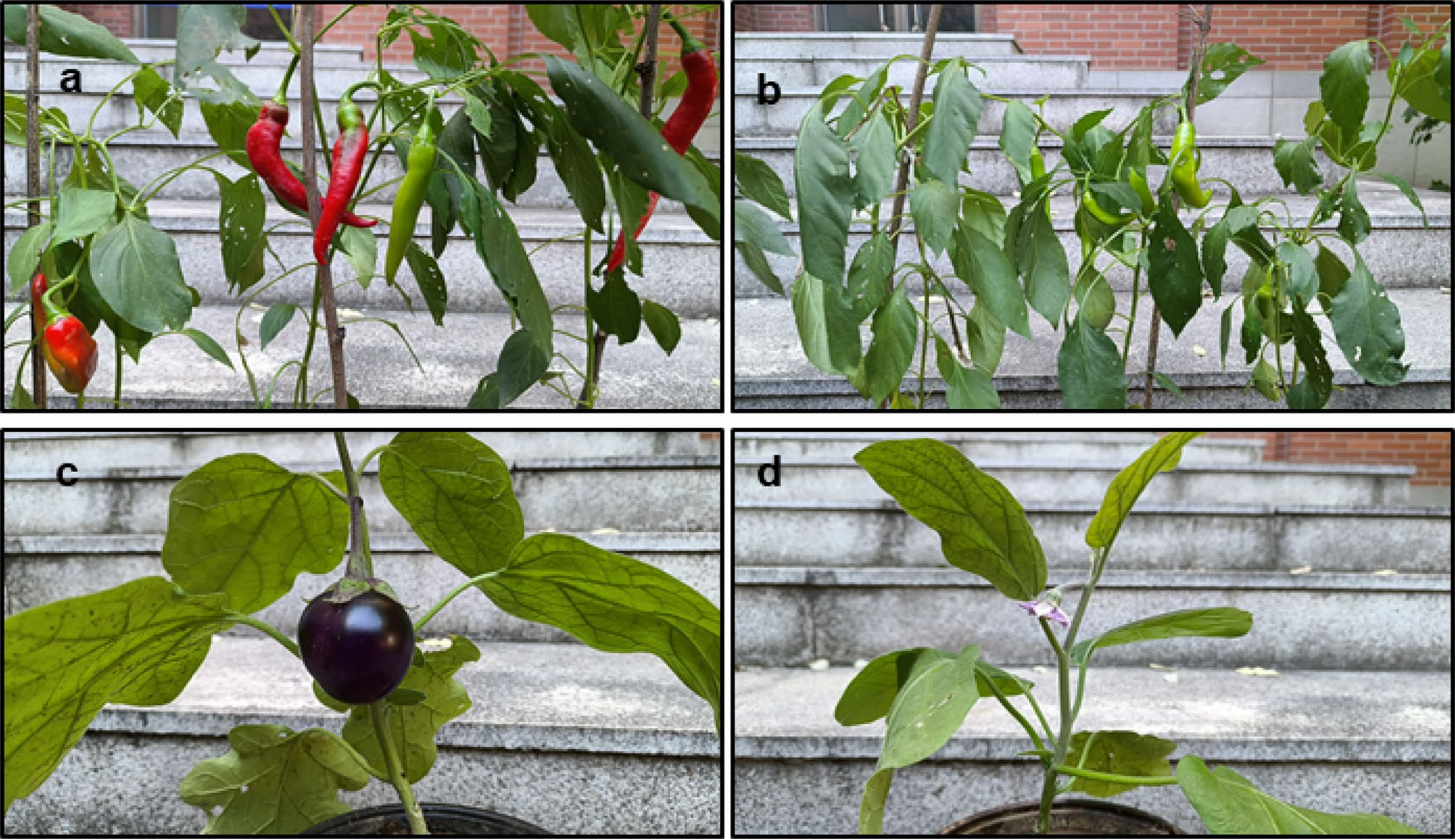
Analysis of plant growth characterized by the flowering and fruiting process at the end of the 3 months. (**a**) Chili grown in soil with effluent, and its control without effluent (**b**); (**c**) brinjal grown in soil with effluent and its corresponding control grown without effluent (**d**).

At the end of the 3rd month, the plants in soil + electrohydrogenesis effluent generated more fruit with big size than the control plants (see Fig. 8), but the tomato (*Solanum lycopersicum* L.) didn’t generate fruits in both protocols at that time probably due to the high weather temperature that inhibitory affected its continuous growth, as previously reported that tomato species are generally so sensitive to temperature change^28,29^. The final yield was evaluated in terms of the size and number of fruits per cultivated plant. Chili cultivated in soil with MEC effluent generated 3 fruits/plant and its corresponding control without effluent produced only 1 fruit/plant. The chili fruit size in soil + effluent was 16 cm, approximately 18.7% higher than its corresponding control. Moreover, at the time of collecting data, the brinjal plant cultivated in soil with MEC effluent generated brinjal fruits whereas its corresponding cultivated without electrohydrogenesis effluent started flowering (see Fig. 8). These further indicate the significant contribution of the electrohydrogenesis effluent in speeding up the plant growth. Herein, the electrohydrogenesis left-over residues have notably improved the soil quality and significantly promoted the plants’ phenology characterized by plant growth, the generation of new leaves, flowering, and the production of fruits.

## Discussion

Biofertilizers are generally defined as various microbial cells with the capacity of enhancing soil fertility. Utilizing microorganisms as biofertilizers could be a promising alternative to costly chemical fertilizers in the agricultural sector owing to their massive potential in promoting crop production and food safety. In this current work, upon completion of the conversion of the lignocellulosic biomass into biogas via a DF-MEC integrated system, the fermentation effluent was collected and analyzed for its possibility of being considered as a potential biofertilizer (see Fig. 1). Interestingly a number of the well-known microorganisms with remarkable impacts to boost soil fertility and plant growth were abundantly found in the electrohydrogenesis left-over residues and are deeply discussed in this work.

Among them, the nitrogen-fixing microorganisms include *Azospirillum* sp., *Rhizobium* sp., *Bradyrhizobium* sp., *Mesorhizobium* sp., *and Beijerinckia* sp. (Fig. 2) which were previously reported for their noticeable contribution in plant nitrogen-fixation mechanisms^25^. For instance, *Azospirillum* sp. (0.11 ± 0.02%) was found in the fermentation residues from DF-MEC integrated reactors, and this bacterial genus is generally known as model plant growth-promoting bacteria. It has proven to have the capacity of colonizing various plant species and remarkably promotes their growth, development, and productivity under normal field conditions^24^. Biological nitrogen fixation is its main mechanism, which was evidenced by the inoculation process^30^. A previously published work reported that inoculating *Azospirillum* sp. in dryland crops increased grain yield by 14% in winter, increased by 9.5% for summer cereals, and augmented by 6.6% for legumes. The inoculation of this bacterial genus into the plant remarkably promotes the nitrogen content both in shoots and grains. Moreover, it has the potential to balance the needed doses of nitrogen fertilization for various plant/crop species; promote the development of roots and favorise the uptake of essential macroelements for plant growth including N, P, and K^31,32^. Generally, those aforementioned nitrogen-fixing microorganisms found in this current bioelectrohydrogenesis residues were reported to convert that atmospheric nitrogen to ammonia by using an enzymatic complex known as nitrogenase^25^. The latter consists of dinitrogenase reductase (with Fe as its cofactor) and dinitrogenase (with Fe and Mo as its cofactors)^33^ (see Fig. 3).

Moreover, the other group of microorganisms including *Pseudomonas* sp., *Bacillus* sp., *Rhodococcus* sp., *Rhizobium* sp., *Azospirillum* sp., *Microbacterium* sp. *Achromobacter* and *Flavobacterium* also detected in the electrohydrogenesis effluent, demonstrated great potential to solubilize insoluble phosphorus into soluble forms that could easily be used by the plant to promote its development. Therefore, residues such electrohydrogenesis effluent containing such microorganisms are on the top list as sustainable biofertilizers owing to their capacity to provide an adequate assimilable amount of phosphorus to various plants/ or crops. Besides providing phosphorus in soluble form to the plants, phosphate-solubilizing bacteria also augment plant growth by stimulating the efficiency of biological nitrogen fixation by nitrogen-fixing microorganisms^25,34^ (Fig. 4).

The phytohormone-producing microorganisms namely *Mycobacterium* sp.*, Allorhizobium* sp., *Pararhizobium* sp., *Paenibacillus* sp., *Bradyrhizobium* sp., *Rhizobium* sp.*, Acinetobacter* sp., and *Azospirillum* sp. were also abundantly found in the electrohydrogenesis effluent. They involve in indole-3-acetic acid/indole acetic acid (IAA) manufacturing, which promotes various plant physiological mechanisms such as plant cell division and differentiation; it induces seed and tuber germination; promotes the development of roots, and initiates both lateral and adventitious root formation. It generally controls vegetative growth processes and mediates responses to light, gravity, and fluorescence. It remarkably influences photosynthesis and pigment formation; governs most of the biosynthesis of various metabolites and supports resistance to stressful environmental conditions^35^. Moreover, it has been shown that those aforementioned rhizobia strain significantly increase the plant leaves surface areas, and control the stomates-adequate opening and closing time to ensure high photosynthetic plants’ rate, and efficiency in water utilization^36^. Additionally, *Azospirillum* sp*.* produces the most extensively reported growth promoters including cytokinins, gibberellins, ethylene, abscisic acid and salicylic acid, auxins, vitamins such as niacin, pantothenic acid, and thiamine, which play an important role in growth promotion^37^ (see Fig. S1).

Furthermore, the collected electrohydrogenesis residues contained heavy metals-bioremediating microorganisms that mainly include *Rhizobium* sp., *Mesorhizobium* sp., *Bradyrhizobium* sp., *Pseudomonas* sp., and *Achromobacter* sp. The common mechanisms exerted by those bacteria are mainly associated with the production of 1-aminocyclopropane-1-carboxylate deaminase which is known to lessen or inhibit the accumulation of stress-induced hormone (ethylene) induced by heavy metal toxicity in plants^38^. Another mechanism is the production of microbial siderophores which alleviate the stress obtruded on plants or crops by creating steady complexes containing toxic heavy metals namely Pb, Cu, Zn, Cd, and Cr, especially for *Chryseobacterium* sp.^39,40^. Those bacterial groups were also reported to reduce metal toxicity by heavy metals biosorption on bacterial cells via metabolism-dependent or independent approaches^41^. Beside promoting plant growth, some microorganisms such as *Azospirillum* sp., *Bacillus* sp., Enterobacter sp., *Gordonia* sp., *Paenibacillus* sp., *Pseudomonas* sp., and *Actinomycetes* sp., are all found in the electrohydrogenesis residues in this current work, and were also reported to hold the ability to reduce pesticide toxicity via enzymatic biodegradation process^25,42^ (Fig. 2). The most-reported key enzymes involved in pesticides degradation include esterases, hydrolases, mixed-function oxidases, and the glutathione S-transferases system through various metabolic reactions such oxidation, hydrolysis, amino group addition to a nitro group, nitro group reduction to an amino group, dehalogenation, ring cleavage, and replacement of sulfur with an oxygen^43^. Therefore, residues from the electrohydrogenesis process could be a potential biofertilizer as they hold a promising approach to alleviating pesticide contamination in soil in an eco-friendly and sustainable manner.

Apart from the plant-growth-promoting microorganisms found in the electrohydrogenesis residues, macro-nutrients that are comparatively needed in large amounts for plant growth promotion were also detected and discussed (Fig. 5). Nitrogen has the capability of promoting the vegetative growth of plants. It significantly contributes to the starch manufacturing in the plant leaf, provides amino acids as raw materials for protein synthesis, and thence enhanced crop production^44^. Phosphorus, one of the primary elements for plant growth, is the key component of cellular membranes, nucleic acids, and metabolic enzymes. Hence, it plays a major role in the various cellular process such as carbohydrate metabolism, energy production, signaling, photosynthesis, and redox-homeostasis. For example, it intervenes as a potential activator for more than sixty plant enzymes, balances water content, and acted as a salt-reducing agent in plants^44^. Similarly, potassium is also a water-regulating element in plants, an enzyme activator, and takes part in photosynthetic metabolic processes which thus lead to promoted plant development. Sulfur is an essential element that is involved in amino acid manufacturing, especially methionine and cysteine. It is a major component of a cofactor/prosthetic group in the Fe–S center, S-adenosyl methionine, thiamine, and various metabolites^44,45^. Sulfur deficiency in plants turns the leaves pale yellow as for N deficiency. The residues also enriched various micro-nutrients such as Zinc, Iron, etc. Zn was reported to drive numerous plant physiological processes including carbohydrate metabolism, cell proliferation, and P-Zn interactions^46^; whereas Iron (Fe) was reported as an indispensable element for the chlorophyll synthesis and preserves the chloroplast structure; however, they are both required at a very little concentration (Fig. 5).

Consistently, the direct application of the collected electrohydrogenesis left-over residues as fertilizer in this study conspicuously promoted the growth and development of the three selected plants namely tomato (*Solanum lycopersicum* L.), chilli (*Capsicum annuum* L.), and brinjal (*Solanum melongena* L.) (see Figs. 6, 7). Moreover, the addition of the MEC effluent speeded up the plant phenology characterized by flowering and fruit-generating process owing to the availability of the plant growth promoting rhizobia (PGPR), nitrogen-fixing microorganisms, phosphate-solubilizing microorganisms, phytohormone-producing microorganisms, heavy metals-bioremediating microorganisms, and sufficient amount of micro-and macronutrients in the effluent (see Fig. 8).

Therefore, the detected level of micro and macroelements and the dominance of microorganisms with fertilizing features in the electrohydrogenesis left-over residues, ensure the safety of using effluent collected from the wheat straw biomass-fed MEC as future biofertilizer for boosting soil quality which thus promotes plant growth as shown in this current research. In that context, recycling bio-electrohydrogenesis left-over residues in agricultural systems will remarkably reduce the use of costly mineral fertilizers, improve soil quality and provide a promising way to control agricultural wastes. However, a further study investigating the level of micro-and macroelements in their bioavailable forms is needed to thoroughly indicate the fertilizing value of electrohydrogenesis left-over residues after biodegradation. Moreover, since the MEC effluent composition primarily depends on the type of substrate used, therefore further research employing MEC effluent obtained from reactors operated with lignocellulosic agricultural wastes other than wheat straw biomass as biofertilizer is needed to confirm the efficiency of using such residues from the overall agricultural wastes for improving soil fertility and plant development.

## Supplementary Information

Supplementary Figure S1.

## Data Availability

The datasets generated and analyzed during the current study are available in the NCBI short read archive (SRA) under the Bioproject accession number PRJNA843580, with Biosample accessions SAMN28748668, SAMN28748669, SAMN28748670, and SAMN28748671.

## References

[1] Holm-Nielsen JB, Al Seadi T, Oleskowicz-Popiel P (2009). The future of anaerobic digestion and biogas utilization. Bioresour. Technol..

[2] Möller K, Stinner W (2009). Effects of different manuring systems with and without biogas digestion on soil mineral nitrogen content and on gaseous nitrogen losses (ammonia, nitrous oxides). Eur. J. Agron..

[3] Stinner W, Möller K, Leithold G (2008). Effects of biogas digestion of clover/grass-leys, cover crops and crop residues on nitrogen cycle and crop yield in organic stockless farming systems. Eur. J. Agron..

[4] Alburquerque JA, de la Fuente C, Bernal MP (2012). Chemical properties of anaerobic digestates affecting C and N dynamics in amended soils. Agric. Ecosyst. Environ..

[5] Odlare M (2011). Land application of organic waste—Effects on the soil ecosystem. Appl. Energy.

[6] Verdi L (2019). Does the use of digestate to replace mineral fertilizers have less emissions of N2O and NH3?. Agric. For. Meteorol..

[7] Tambone F (2010). Assessing amendment and fertilizing properties of digestates from anaerobic digestion through a comparative study with digested sludge and compost. Chemosphere.

[8] Przygocka-Cyna K, Grzebisz W (2018). Biogas digestate—Benefits and risks for soil fertility and crop quality—An evaluation of grain maize response. Open Chem..

[9] Makdi M, Tomcsik A, Orosz V (2012). Digestate: A New Nutrient Source—Review.

[10] USDA (2007). Nitrogen Efficiency and Management.

[11] Doyeni MO, Stulpinaite U, Baksinskaite A, Suproniene S, Tilvikiene V (2021). The effectiveness of digestate use for fertilization in an agricultural cropping system. Plants.

[12] Malusa E, Vassilev N (2014). A contribution to set a legal framework for biofertilisers. Appl. Microbiol. Biotechnol..

[13] Vessey JK (2003). Plant growth promoting rhizobacteria as biofertilizers. Plant Soil.

[14] Mazid M, Khan TA (2014). Future of bio-fertilizers in Indian agriculture: An overview. Int. J. Agric. Food Res..

[15] Häfner F, Ruser R, Claß-Mahler I, Möller K (2021). Field application of organic fertilizers triggers N2O emissions from the soil N pool as indicated by 15N-labeled digestates. Front. Sustain. Food Syst..

[16] Li X-H, Liang D-W, Bai Y-X, Fan Y-T, Hou H-W (2014). Enhanced H2 production from corn stalk by integrating dark fermentation and single chamber microbial electrolysis cells with double anode arrangement. Int. J. Hydrogen Energy.

[17] Yan X (2021). Enhanced straw fermentation process based on microbial electrolysis cell coupled anaerobic digestion. Chin. J. Chem. Eng..

[18] Walter WG (1961). Standard methods for the examination of water and wastewater (11th ed.). Am. J. Public Health Nations Health.

[19] Alburquerque JA (2012). Agricultural use of digestate for horticultural crop production and improvement of soil properties. Eur. J. Agron..

[20] Liu W (2016). Microbial electrolysis contribution to anaerobic digestion of waste activated sludge, leading to accelerated methane production. Renew. Energy.

[21] Caporaso JG (2010). QIIME allows analysis of high-throughput community sequencing data. Nat. Methods.

[22] Yang J, Yu Z, Wang B, Ndayisenga F (2021). Gut region induces gastrointestinal microbiota community shift in Ujimqin sheep (*Ovis aries*): From a multi-domain perspective. Environ. Microbiol..

[23] Edgar RC (2010). Search and clustering orders of magnitude faster than BLAST. Bioinformatics.

[24] Bashan Y, De-Bashan LE (2010). How the plant growth-promoting *Bacterium azospirillum* promotes plant growth—A critical assessment. Adv. Agron..

[25] Mahanty T (2017). Biofertilizers: A potential approach for sustainable agriculture development. Environ. Sci. Pollut. Res. Int..

[26] Shinwari KI (2015). Application of plant growth promoting rhizobacteria in bioremediation of heavy metal polluted soil. Asian J. Multidiscipl. Stud..

[27] Risberg K, Cederlund H, Pell M, Arthurson V, Schnurer A (2017). Comparative characterization of digestate versus pig slurry and cow manure—Chemical composition and effects on soil microbial activity. Waste Manag..

[28] Alsamir M, Mahmood T, Trethowan R, Ahmad N (2021). An overview of heat stress in tomato (*Solanum lycopersicum* L.). Saudi J. Biol. Sci..

[29] Guo T (2022). Heat stress mitigation in tomato (*Solanum lycopersicum* L.) through foliar application of gibberellic acid. Sci. Rep..

[30] Okon Y, Heytler PG, Hardy RW (1983). N(2) fixation by *Azospirillum brasilense* and its incorporation into Host *Setaria italica*. Appl. Environ. Microbiol..

[31] Kapulnik Y, Kigel J, Okon Y, Nur I, Henis Y (1981). Effect of Azospirillum inoculation on some growth parameters and n-content of wheat, sorghum and panicum. Plant Soil.

[32] Cassán F, Diaz-Zorita M (2016). *Azospirillum* sp. in current agriculture: From the laboratory to the field. Soil Biol. Biochem..

[33] Smith BE, Richards RL, Newton WE (2004). Catalysts for Nitrogen Fixation: Nitrogenases, Relevant Chemical Models, and Commercial Processes.

[34] Mohammadi K, Sohrabi Y (2012). Bacterial Biofertilizers for Sustainable Crop Production: A Review.

[35] Shailendra Singh GG (2015). Plant growth promoting rhizobacteria (PGPR): Current and future prospects for development of sustainable agriculture. J. Microb. Biochem. Technol..

[36] Mia MAB, Shamsuddin ZH (2010). Nitrogen fixation and transportation by rhizobacteria: A scenario of rice and banana. Int. J. Bot..

[37] Raffi MM, Charyulu PBBN (2021). Azospirillum-Biofertilizer for Sustainable Cereal Crop Production: Current Status.

[38] Singh RP, Shelke GM, Kumar A, Jha PN (2015). Biochemistry and genetics of ACC deaminase: A weapon to "stress ethylene" produced in plants. Front. Microbiol..

[39] Radzki W (2013). Bacterial siderophores efficiently provide iron to iron-starved tomato plants in hydroponics culture. Antonie Van Leeuwenhoek.

[40] Saha R, Saha N, Donofrio RS, Bestervelt LL (2013). Microbial siderophores: A mini review. J. Basic Microbiol..

[41] Dary M, Chamber-Perez MA, Palomares AJ, Pajuelo E (2010). "In situ" phytostabilisation of heavy metal polluted soils using Lupinus luteus inoculated with metal resistant plant-growth promoting rhizobacteria. J. Hazard Mater..

[42] Shaheen, S. & Sundari, S. K. *Exploring the Applicability of PGPR to Remediate Residual Organophosphate and Carbamate Pesticides Used in Agriculture Fields* (2013).

[43] Laura, M., Snchez-Salinas, E., Dantn Gonzlez, E. & Luisa, M. *Pesticide Biodegradation: Mechanisms, Genetics and Strategies to Enhance the Process* (2013).

[44] Kumar S, Kumar S, Mohapatra T (2021). Interaction between macro- and micro-nutrients in plants. Front. Plant Sci..

[45] Koprivova A, Kopriva S (2014). Molecular mechanisms of regulation of sulfate assimilation: First steps on a long road. Front. Plant Sci..

[46] Rehman H-U, Aziz T, Farooq M, Wakeel A, Rengel Z (2012). Zinc nutrition in rice production systems: A review. Plant Soil.

