# Supplementary material for: Using bioelectrohydrogenesis left-over residues as a future potential fertilizer for soil amendment
Source: Sci Rep. 2022 Oct 22;12:17779. doi: 10.1038/s41598-022-22715-x (PMC9588085; doi:10.1038/s41598-022-22715-x)
Supplement: Supplementary file 1 — Supplementary Figure S1. [file 41598_2022_22715_MOESM1_ESM.docx]

**Using bioelectrohydrogenesis left-over residues as a future potential fertilizer for soil amendment**

Fabrice Ndayisenga ^1,2,3^, Zhisheng Yu ^1, 2, 3*^, Bobo Wang^1, 3^, Jie Yang ^1, 2, 3^, Gang Wu ^1, 4^, Hongxun Zhang ^1, 2, 3^

^1^College of Resources and Environment, University of Chinese Academy of Sciences, 19 A Yuquan Road, Beijing 100049, P.R. China.

^2^Binzhou Institute of Technology, Weiqiao-UCAS Science and Technology Park, Binzhou City 256606, Shandong Province, P.R. China

^3^RCEES-IMCAS-UCAS Joint-Lab of Microbial Technology for Environmental Science, Beijing 100085, China

^4^State Key Laboratory of Urban and Regional Ecology, Research Center for Eco-Environmental Sciences, Chinese Academy of Sciences, Beijing, 100085, China

**^*^ Author for correspondence:** Prof. Dr. Zhisheng Yu, E-mail: yuzs@ucas.ac.cn

**Supplementary Information**

3Pages including cover page

1Figure

**Figure S1.** Schematic diagram illustrating the positive short-term effects of seeds and seedlings inoculation with *Azospirillum* sp.


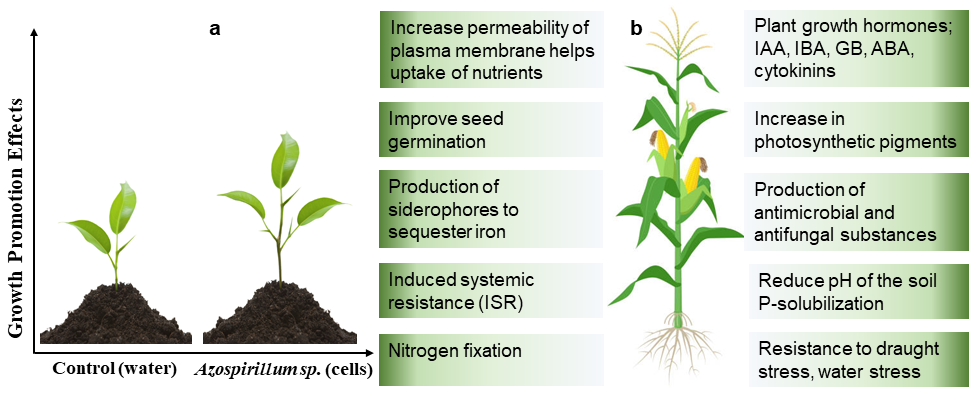


**Note:** (a); where the control (free of plant growth-promoting bacteria) never induced growth promotion on seeds or seedlings, and the inoculation with *Azospirillum* sp*.* (phytohormones producer rhizobacteria) induces a higher growth promotion response on seeds or seedlings; and (b) indicate the different modes of action of *Azospirillum* on plant growth. (Figure was adapted from ^1,2^.

**References**

1. Raffi, M.M. & Charyulu, P.B.B.N. Azospirillum-biofertilizer for sustainable cereal crop production: Current status. 193-209 (2021).

2. Cassán, F. & Diaz-Zorita, M. Azospirillum sp. in current agriculture: From the laboratory to the field. *Soil Biology and Biochemistry* **103**, 117-130 (2016).
